# Treatment of Unicompartmental Cartilage Defects of the Knee with Unicompartmental Knee Arthroplasty, Patellofemoral Partial Knee Arthroplasty or Focal Resurfacing

**DOI:** 10.3390/life11050394

**Published:** 2021-04-27

**Authors:** Bernhard Springer, Friedrich Boettner

**Affiliations:** 1 Department of Orthopedic and Trauma Surgery, Vienna General Hospital, Medical University of Vienna, Waehringer Guertel 18–20, 1090 Vienna, Austria; bernhard.springer@meduniwien.ac.at; 2 Adult Reconstruction and Joint Replacement Division, Hospital for Special Surgery, 535 E 70th Street, New York, NY 10021, USA

**Keywords:** unicompartmental knee arthroplasty, patellofemoral joint replacement, focal resurfacing, focal chondral defects, OA of the knee

## Abstract

Focal chondral defects are common lesions of the articular cartilage. They are predominantly found on the medial femoral condyle and often progress to osteoarthritis of the knee. Various conservative treatment options are available. The conservative treatment might reduce pain and delay the progress of degenerative processes. However, restoration of the articular cartilage cannot be accomplished. If the conservative treatment fails unicompartmental arthroplasty, patellofemoral joint replacement or focal resurfacing are reasonable options to postpone total knee arthroplasty. A careful patient selection before surgery is crucial for all three treatment options. The following overview reports indications and outcomes of medial partial knee replacement, patellofemoral partial knee replacement, and focal resurfacing treatment options for focal chondral defects.

## 1. Introduction

Focal chondral defects (FCD) are common disruptions of the continuity of the cartilage layers. They might occur on every articular cartilage surface of the body; however, chondral defects are predominantly found on the medial femoral condyle [1,2]. Focal chondral defects of the hip predominately occur as a result of trauma or in patients with hip dysplasia or femoroacetabular impingement [3,4]. However, generally FCD of the hip are rare. The current literature hardly provides any outcome data following cartilage repair of FCD of the hip joint. The arthroscopic access to these defects is difficult and open procedures are associated with a significant morbidity [5]. As a result, surgical intervention is hardly ever attempted for FCD of the hip. Several treatment options are available for FCD of the talus. For younger patients with isolated localized defects and without ankle deformity, biological repair techniques are the preferred therapy options [6]. Isolated symptomatic defects of the talus in middle-aged patients are difficult to treat, as patients might neither be suitable for biological repair treatments nor for replacement/fusion surgeries [7].

Due to low vascular and neural supply of articular cartilage, the regenerative potential of FCD is limited [8,9]. Most cartilage lesions do not result in clinical symptoms [10]. Patients that present with clinical symptoms most often complain of joint effusions, joint pain or loss of flexibility. However, the majority of patients with clinical symptoms describe an unspecific knee pain. Frank et al. [11] reported more severe clinical symptoms when the defect was located in the weight-bearing zone of the knee.

Previous studies have shown that FCD are an important risk factor for an early onset of osteoarthritis (OA) of the knee and, therefore, early detection is crucial [12,13,14,15]. Cicuttini et al. [16] showed that even asymptomatic middle-age patients with small defects of mild severity had large reductions of the cartilage volume in the medial and lateral compartment of the knee.

Various treatment options are available for FCD; however, it is unclear which treatment has the best potential to restore a functional articular-cartilage-like tissue [10]. Factors that should be taken into consideration include: defect size, defect location, defect depth, mechanical knee alignment and knee stability, as well as patient demand [17]. In addition, patient-specific systemic factors, including age, activity level, body mass index (BMI), and sex need to be considered [18].

Non-operative treatment options aim at the reduction of clinical symptoms and the delay of progression of the degenerative processes [10]. They include physical therapy, rest during the acute injury phase, ice, weight reduction, unloader braces, muscle strengthening or anti-inflammatory medication. If pain relief is not achieved with these options, intraarticular corticosteroid injections, injection of hyaluronate derivates or other available bioactive injectables might be considered as a temporary solution before indicating surgical interventions [19,20]. Non-operative treatments, however, can only reduce pain, but restoration of articular cartilage cannot be accomplished [17].

Focal chondral defects International Cartilage Repair Society (ICRS) grade 3 or 4 should be addressed surgically [21]. For smaller defects (<2–4 cm^2^) in patients <40 years various surgical treatment options such as local debridement, chondroplasty, marrow stimulation techniques (e.g., microfracture, drilling) or osteochondral autograft/allograft transfers are reasonable treatment options [22,23]. In young patients with defects >2.5 cm^2^ autologous chondrocyte transplantation is the preferred treatment option, as promising long-term results were achieved in cases with larger defects [24,25]. Gilat et al. [26] showed significantly improved clinical results after osteochondral allograft transplantation in larger FCD of the femoral condyles with 5-year survival rates of 86.2% and 10-year survival rates of 81.8%, respectively. Another randomized study compared microfracture with autologous chondrocyte implantation and found that one third of patients in both groups had radiographic evidence of OA of the knee at the 5-year follow up [27]. Especially, tibial FCD and concomitant lesions of the meniscus are prognostic factors for accelerated progression of the OA of the knee [28]. Kreuz et al. [29] showed that 18 months after microfracture treatment a deterioration begins, which is significantly worse in patients over 40 years of age. Higher failure rates were also found in patients ≥40 years after osteochondral allograft transplantation [30].

In up to 47% of patients over 60 years of age, FCD progresses to diffuse OA of the knee [31,32]. For these particular patients, partial or total joint arthroplasty is usually the re-commended treatment option [22].

The following overview reports indications and outcomes of medial partial knee replacement, patellofemoral partial knee replacement, and focal resurfacing treatment options for focal chondral defects.

## 2. Focal Chondral Defects and Unicompartmental Knee Arthroplasty

### 2.1. Indications for Unicompartmental Knee Arthroplasty

Indications and contraindications for unicompartmental knee arthroplasty (UKA) are discussed controversially [33]. To achieve good long-term results, an adequate patient selection and careful preoperative evaluation is crucial [34,35].

In 1989, Kozinn and Scott [36] presented a selection criteria for medial UKA. They suggested performing medial UKA only in patients who are:

- over 60 years old;
- weigh less than 82 kg;
- have isolated medial compartment OA;
- have no patellofemoral OA;
- no lateral joint tenderness;
- no anterior knee pain;
- low physical demands;
- a varus deformity of under 10°;
- a flexion contracture of under 5°;
- intact ligaments and no inflammatory conditions.

Up to today, many surgeons stick to these suggested selection criteria [37]. Over the last 30 years, indications for medial UKA were extended based on published evidence. Even if the primary indication for medial UKA is still end-stage anteromedial OA of the knee [38], it also became a reasonable treatment option for other pathologies such as avascular osteonecrosis [39].

Full thickness cartilage defects in the weight-bearing zone of the medial compartment of the knee are a requirement for medial UKA [36,40] and patients with lateral OA of the knee should not be scheduled for medial UKA [41]. However, previous studies have shown that the presence of lateral osteophytes is not associated with more-advanced histological cartilage degeneration, lower cartilage volume, biomechanically weaker cartilage or diminished cartilage thickness in the lateral compartment [42,43]. Therefore, lateral osteophytes should not be considered as a contraindication for medial UKA.

Intact cartilage in the lateral compartment is another essential requirement for medial UKA and the progression of lateral OA is a common cause for failure of medial UKA [40]. A previous study evaluated the impact of white blood cell (WBC) count in the synovial fluid on biomechanical cartilage properties [44]. They found that the cartilage quality in the lateral compartment was worse in knees with an elevated WBC count compared to knees with a low WBC count.

The current literature provides several studies that evaluated radiographic findings to identify lateral compartment OA. It has been shown that a positive tibial spine sign on AP radiographs of the knee does not indicate cartilage defects in the central area of the lateral compartment [45].

Furthermore, Waldstein et al. [46] showed that valgus stress radiographs of the knee are not suitable to identify lateral compartment OA with absolute certainty.

Another essential requirement for medial UKA is a functional sufficient anterior cruciate ligament (ACL) [40,47]. High numbers of tibial component loosening were reported for UKA in patients with an insufficient ACL [48,49]. In younger, more active patients, combined UKA with ACL reconstruction might be a treatment option [47,50]. While this is a technically demanding procedure, previous studies showed encouraging results [51,52].

For UKA, patient selection is crucial and various radiographs should be taken preoperatively. Hamilton et al. [53] provided a radiographic decision aid to identify patients that are suitable for UKA. Lateral radiographs of the knee can be used preoperatively to rule out posterior tibial erosion and, therefore, ACL insufficiency [54].

In uncertain cases, preoperative magnetic resonance imaging (MRI) is recommended. The percentage of full-thickness cartilage posteriorly on the medial tibial plateau should be evaluated on the MRI. Less than 14% intact posterior cartilage indicates wear patterns that are associated with a functionally insufficient ACL [54].

As mentioned earlier, Kozinn and Scott postulated that varus alignment must be <10° to perform UKA surgery. However, medial UKA is still indicated if the intra-articular deformity is correctable after removal of medial osteophytes [53]. The correctability of deformity can be evaluated reliably using preoperative valgus stress radiographs [55]. Another radiographic finding that is associated with varus OA of the knee and more severe varus deformity, is the “coronal tibiofemoral subluxation” (CTFS) which is evaluated on AP radiographs of the knee [56]. A recent study found that a CTFS of ≥6 mm and a varus deformity of ≥10° are indicators for an insufficient ACL [34]. Therefore, both factors should be taken into consideration before scheduling a patient for UKA and medial UKA should not be performed if these values are exceeded.

### 2.2. Results

One third of patients with focal chondral defects in the medial compartment of the knee need a joint replacement surgery within 10 years [28]. The differentiation between symptoms of FCD or OA is difficult. A recent study found that patients that are scheduled for joint replacement surgery due to OA of the knee complain significantly more often about medial- sided pain, swelling and pain while fully extending the knee, standing upright or rising from a seated position [57]. However, the current literature does not provide data on the clinical outcome of patients with FCD of the knee that underwent UKA surgery. Further research is needed to shed light on the outcome of these particular patients. In cases of medially located chondral defects that progress to anteromedial OA, UKA is a well-accepted and effective long-term treatment option [58].

## 3. Focal Chondral Defects and Patellofemoral Joint Replacement

### 3.1. Indications for Patellofemoral Joint Replacement

Patellofemoral joint replacement (PFJR) is indicated in cases with isolated degeneration or deformity in the patellofemoral joint due to dysplasia or instability or FCD that progressed to OA in the patellofemoral joint [59]. Especially young and active patients <50 years with higher demands might be considered for PJFR. While small lesions can be replaced with a small resurfacing implant, larger areas of osteoarthritis usually require patellofemoral replacement including a patella component [60].

In patients with tibiofemoral OA, PFJR is contraindicated [61]. Restricted range of motion, chronic knee pain, inflammatory diseases of the knee or instability of the knee are also considered a contraindication [60].

Most isolated osteochondral defects in the patellofemoral joint are caused by a mechanical mismatch of patellofemoral joint components resulting in increased loads on the patellofemoral cartilage [62]. Dysplasia or malalignment might contribute to the mismatch. In these cases, a combined approach is recommended [60,63].

In patients with a pronounced dysplasia of the femoral trochlea, PFJR with combined reconstruction of the medial patellofemoral ligament can be considered to prevent the patella from subsequent luxation [64]. Lateral position of the tibial tuberosity might cause maltracking of the patella. Simultaneous transfer of the tibial tuberosity is a treatment option in these cases to ensure that loading forces affect the patella medially and laterally equally. Maltracking can also to some degree be corrected by increasing the external rotation of an onlay femoral component. Combined PFJR and high tibial osteotomy or distal femur osteotomy might be used in patients with a deformity >5° varus or valgus [65]. However, the authors do not favor combined approaches since increased deformities are often a predictor for progression of the OA in the medial or lateral compartment and the outcome of a later total knee replacement is usually compromised by a prior femoral or tibial osteotomy [66,67].

For PFJR two different types of implants are available: Onlay-designs and Inlay-designs [61,68]. Onlay-implants replace the entire patellofemoral articular surface and produce a newly shaped trochlea. They are suitable for larger defects. The potential risk of “overstuffing” (narrowing the patellofemoral joint space) is a disadvantage of these implant types [61]. Inlay-implants only replace the affected area of articular surface. The implant is adjusted to the shape of the trochlea. Using Inlay-implants, overstuffing cannot occur [61]. Intraoperatively, a proper alignment of the prosthesis and soft-tissue balancing are crucial to optimize results and avoid patella-maltracking [69].

### 3.2. Results

The benefit of PFJR has been debated due to its high failure rates [70]. Revision rates were also high—especially when early Inlay-designs of patellofemoral prostheses were used [71]. As a result, total knee arthroplasty (TKA) has been the preferred treatment option for patellofemoral osteoarthritis regardless of the functional advantages a PFJR might provide compared to TKA [72,73]. When comparing modern designs of PFJR and TKA, no differences in the number of complications or revisions have been reported [72].

Careful patient selection prior to PFJR is crucial. A recent study showed similar patient-reported outcomes after second-generation PFJR and TKA if patients were selected appropriately [74]. Dejour et al. [75] postulated that early revision surgeries due to OA progression only occurred when patients were selected improperly. They also showed that PFJR should be restricted to patients that developed patellofemoral OA due to instability or maltracking of the patella and that it should not be used in patients with degenerative patellofemoral OA.

When comparing Inlay-designs with Onlay-designs in a matched-paired cohort, no differences were found between the two designs and both designs led postoperatively to significantly better functional outcome scores [76]. The only observed difference was that the progression of tibiofemoral OA occurred significantly more often in patients with an Onlay-design prosthesis.

Unnithan et al. [77] evaluated the outcome of six patients that underwent combined PFJR and osteochondral autograft transfer system (OATS) and found encouraging results. Only one patient needed a revision surgery due to progressive symptoms.

In cases of failed Inlay-prosthesis, it is a reasonable option to use an Onlay-prosthesis for revision surgery [71].

A recent retrospective study compared the outcome of patients with asymptomatic patellofemoral FCD undergoing UKA only versus undergoing UKA and concomitant PFJR with an Inlay-design prosthesis [78]. There was no benefit for patients who underwent the combined procedure, showing that even pronounced trochlear defects can be ignored during UKA if patients were asymptomatic preoperatively.

The current literature does not provide long-term outcomes of PFJR and further research is needed to confirm the encouraging short-term results also in the long-term.

## 4. Focal Chondral Defects and Focal Resurfacing

Symptomatic focal chondral lesions of the femoral condyle occur frequently in patients between 40–60 years [1,2]. The current literature provides hardly any data on treatment of middle-aged patients with FCD and failed biological treatment or patients with FCD who are not suitable for biological treatment options [79,80].

For focal full-thickness cartilage defects of the femoral condyle, a focal prosthetic inlay resurfacing is another possible treatment option when biological treatments have failed and total knee arthroplasty is not yet justified [81].

The indications for these implants are as follows [82]: Patients > 35 years, isolated osteochondral or full-thickness cartilage defects in weight-bearing areas of the femoral condyle (preferably the medial femoral condyle), intact ligaments, full range of motion, normal contralateral knee joint. Age > 65 years, varus or valgus alignment > 7°, BMI > 35 kg/m^2^ or cartilage damage in another compartment of the knee should be considered as contraindications [82].

As patients with focal metallic inlay resurfacing had significantly improved knee function and also significantly less pain, these implants were considered as an effective treatment option [80]. However, inconsistent results were published in regard to the OA- progression, ranging from a significant progression within 2 years [83,84] to no progression after 5 years [82].

Becher and Cantiller [22] observed two cases for 12 years. Both patients showed good clinical results and no progression of OA. Other studies showed good results in short-term patient-reported outcome measures, but also reported a high rate of required revision surgery to TKA [83,85].

Similar to UKA and PFJR, a proper preoperative evaluation and careful patient selection is mandatory. Furthermore, it is important to position implants accurately to avoid step offs or increased pressure on the opposite cartilage surface [18,86]. In addition, Malahias et al. [87] reported that Arthrosurface inlay implants were associated with a postoperative effusion. They stated that these procedures should only be used if prior biological treatments failed.

The development of newer customized femoral condyle implants with a combined guiding system streamlined the process to position the implant intraoperatively and helped to ensure an accurate position of the implant [88]. As a result, damage of the opposite cartilage surface can be avoided. Stålman et al. [89] presented the results of the first 10 patients that were treated with the abovementioned customized implant. In a 2-year follow-up, they found a good implant safety and also good to excellent patient-reported outcome measures. However, it is a relatively small cohort and further research is needed to postulate a generalized statement.

## 5. Conclusions

Focal chondral defects of the knee are common lesions that often progress to OA of the knee. Conservative treatment options might reduce pain; however, restoration of the articular cartilage cannot be accomplished in most older patients. If biological treatment options fail, unicompartmental knee arthroplasty, patellofemoral joint replacement or focal resurfacing are reasonable options to postpone total knee arthroplasty. Unicompartmental knee arthroplasty is an accepted and well-evaluated treatment option with good long-term results. Patellofemoral joint replacement and focal resurfacing both showed good short-term results, but long-term results might be not as promising. Nevertheless, careful patient selection is crucial for all three treatment options to achieve best possible results.
